# Integrating Transcriptomics and 3D Spheroid Models Reveals Microenvironment-Dependent Purinergic Modulation in Hepatocellular Carcinoma

**DOI:** 10.3390/metabo16030152

**Published:** 2026-02-25

**Authors:** Arieli Cruz de Sousa, Augusto Ferreira Weber, Vinícius Klain, Juliete Nathali Scholl, Jéssica Marques Obelar Ramos, Natália Baltazar do Nascimento, Maria Luiza Giehl, Renata Kruger Martins, João Vitor Heres, Camila Kehl Dias, Renata Marschner, Fabrício Figueiró, Fátima Costa Rodrigues Guma

**Affiliations:** 1 Laboratory of Differentiation, Proliferation, and Cell Viability, Department of Biochemistry, Institute of Basic Health Sciences, Federal University of Rio Grande do Sul, Porto Alegre 90610-264, RS, Brazil; arieli.sousa@ufrgs.br (A.C.d.S.); jessica.obelar@ufrgs.br (J.M.O.R.); maria.giehl@ufrgs.br (M.L.G.); kruger.martins@ufrgs.br (R.K.M.); joao.heres@ufrgs.br (J.V.H.); fatima.guma@ufrgs.br (F.C.R.G.); 2 Laboratory of Cancer Immunobiochemistry, Department of Biochemistry, Institute of Basic Health Sciences, Federal University of Rio Grande do Sul, Porto Alegre 90610-264, RS, Brazil; augustofweber@mx2.unisc.br (A.F.W.); jnscholl@hcpa.edu.br (J.N.S.); kehl.dias@ufrgs.br (C.K.D.); renata.marschner@ufrgs.br (R.M.); 3 Graduate Program in Biological Sciences, Biochemistry, Institute of Basic Health Sciences, Federal University of Rio Grande do Sul, Porto Alegre 90610-264, RS, Brazil; natalia.baltazar@ufrgs.br; 4 One Health Research Group, Biotechnology Center, Graduate Program in Cellular and Molecular Biology, Federal University of Rio Grande do Sul, Porto Alegre 90610-264, RS, Brazil; vinicius.klain@ufrgs.br

**Keywords:** sorafenib, doxazosin, *NT5E*, hepatic stellate cells, drug resistance

## Abstract

**Background/Objectives**: Dysregulation of purinergic signaling, particularly CD73 overexpression, influences tumor progression, immune evasion, and chemoresistance in hepatocellular carcinoma (HCC). We aimed to characterize the transcriptional landscape of this system, identify prognostic markers, and investigate how the tumor microenvironment modulates pharmacological response to combined sorafenib and doxazosin in 3D spheroid models. **Methods**: We integrated RNA-seq data from The Cancer Genome Atlas—Liver Hepatocellular Carcinoma (TCGA-LIHC) to identify differentially expressed genes, pathway enrichment, gene co-expression networks, prognostic associations, and machine learning-based biomarker selection. Modulation of key targets was assessed in HepG2 and HepG2/LX-2 spheroids treated with sorafenib and doxazosin using qPCR and flow cytometry. **Results**: Transcriptomics revealed dysregulation and network fragmentation. Specifically, analysis of the TCGA cohort indicated that high expression of *ADA*, *NT5E*, and *ADORA1* correlated with poor overall survival. Given the critical role of CD73 in therapy resistance, we evaluated these findings in 3D models. Co-treatment significantly downregulated *NT5E* and *ADORA1* mRNA expression, while *ADORA2A* was specifically reduced in the co-culture setting. For the *ADA*, effect-size analysis revealed a large magnitude of inhibition in HepG2 spheroids. Although flow cytometry showed that high CD73 protein expression remained stable across treatments in co-culture, the combination therapy overcame stromal protection, significantly increasing apoptosis (active caspase-3) in both mono- and co-culture spheroids compared with vehicle and monotherapy. **Conclusions**: We identified a purinergic prognostic signature in HCC and demonstrated that the combination therapy of sorafenib and doxazosin targets the adenosine pathway and specific receptors. We show that the stromal microenvironment sustains CD73 protein expression even under transcriptional inhibition, highlighting the critical role of 3D co-culture models in deciphering therapeutic resistance mechanisms.

## 1. Introduction

Hepatocellular carcinoma (HCC) ranks as the sixth most frequent malignancy and the third leading cause of cancer-related mortality worldwide, with a steadily increasing incidence [1]. Chronic necroinflammation is a primary driver of hepatocarcinogenesis, inducing chromosomal instability and malignant transformation of proliferating hepatocytes. Major risk factors include chronic Hepatitis B and C infections, alcohol abuse, and metabolic disorders such as metabolic-associated steatotic liver disease (MASLD) [2,3]. Furthermore, liver cirrhosis is detected in approximately 90% of HCC patients, highlighting the critical role of fibrotic remodeling in tumor development [4]. Despite surveillance programs, most patients are diagnosed at intermediate or advanced stages, where curative options like resection or transplantation are no longer viable, resulting in a poor prognosis [4,5].

The hepatic tumor microenvironment (TME) plays a pivotal role in disease progression and therapeutic resistance. Hepatic Stellate Cells (HSCs), typically quiescent vitamin A-storing pericytes, undergo activation in response to liver injury, transdifferentiating into proliferative, extracellular matrix-producing myofibroblasts [6]. In the liver TME, activated HSCs are the main source of cancer-associated fibroblasts (CAFs), secreting growth factors (e.g., TGF-β1, PDGF) that fuel tumor growth, invasion, and epithelial–mesenchymal transition (EMT). Consequently, the crosstalk between tumor cells and the stromal compartment acts as a physical and biological barrier against systemic therapies [7,8,9].

However, traditional two-dimensional (2D) monolayer cultures fail to recapitulate this complex spatial architecture and pathophysiology of the in vivo liver tissue. The lack of cell–cell interactions, extracellular matrix (ECM) deposition, and physiological oxygen and nutrients gradients in 2D models often leads to poor predictive value for drug screening [7,8,9,10]. In contrast, three-dimensional (3D) multicellular tumor spheroids (MCTS) serve as a superior preclinical model. By incorporating stromal components like HSCs, co-culture spheroids mimic the physical barriers to drug penetration and the metabolic reprogramming, such as hypoxia-driven adenosine accumulation, that characterizes resistant solid tumors, offering a more reliable platform for evaluating combinatorial therapies [7,8,9,10,11].

Sorafenib, a multikinase inhibitor targeting RAF/MEK/ERK and VEGFR/PDGFR signaling, was the first approved systemic therapy for advanced HCC [12]. While it promotes apoptosis and inhibits angiogenesis, its clinical benefit is often limited by rapid drug resistance, partly driven by stromal interactions and the activation of compensatory survival pathways, most notably the phosphoinositide 3-kinase (PI3K)/Akt signaling axis [13,14]. This scenario underscores the urgent need for novel therapeutic strategies.

Doxazosin, a quinazoline-derived α1-adrenergic blocker used to treat benign prostatic hyperplasia [15,16], has emerged as a promising repurposing candidate. Beyond its antihypertensive effects, doxazosin exhibits antitumor properties in prostate, breast, and glioblastoma models, often via mechanisms independent of α1-adrenoceptors, including the potent inhibition of the PI3K/Akt pathway [17,18,19]. Doxazosin also has an antifibrotic effect, acting directly on HSCs and decreasing their activation markers, such as type I collagen and smooth muscle α-actin [20].

Purinergic signaling consists of extracellular purines and purinergic receptors and modulates cell proliferation, invasion, and immune response during cancer progression [21,22]. The enzymes CD39 (NTPDase-1; *ENTPD1*), CD73 (ecto-5′-nucleotidase; *NT5E*), and ADA (adenosine deaminase; *ADA*) are expressed in immune, tumor, and stromal cells. These enzymes regulate ATP and adenosine levels by hydrolyzing extracellular ATP in multiple steps [23]. In the context of HCC, it has been shown that CD73^+^ CAFs can promote chemoresistance to sorafenib and cisplatin in HCC cells. This indicates that CD73^+^ CAFs are a potential therapeutic target for HCC treatment [24].

Adenosine signaling connects this metabolic landscape to the drug-resistance mechanisms targeted by doxazosin. Recent evidence indicates that the activation of P1 receptors, particularly *ADORA1* (A1R) and *ADORA2A* (A2AR), can directly trigger the PI3K/Akt/mTOR cascade, fueling tumor cell proliferation and inhibiting apoptosis [25,26]. This creates a mechanistic rationale in which the dysregulated purinergic system sustains survival pathways that counteract sorafenib efficacy [24]. Therefore, targeting the purinergic axis could re-sensitize tumor cells to therapy.

Given the high molecular heterogeneity of HCC, targeting a single gene often fails to capture the complexity of therapy resistance. Therefore, mapping the global transcriptional landscape is essential to identify robust prognostic biomarkers and drug targets within specific signaling networks. Integrative approaches that combine large-scale transcriptomic data from patient cohorts, such as The Cancer Genome Atlas (TCGA), with validatable in vitro models provide a powerful translational framework [27,28]. This strategy enables in silico identification of dysregulated pathways and subsequent testing of their pharmacological modulation in biologically relevant systems, thereby bridging the gap between molecular profiling and therapeutic application.

Here, we integrated bioinformatic analysis of the TCGA—Liver Hepatocellular Carcinoma (LIHC) cohort with in vitro 3D spheroid models to investigate the purinergic system in HCC. We hypothesized that the dysregulated purinergic signature correlates with poor prognosis and that the combined treatment with sorafenib and doxazosin could remodel this signaling by targeting the adenosine–PI3K axis. Using HepG2 and HepG2/LX-2 spheroids, we investigated whether the stromal microenvironment protects the tumor by altering purinergic targets and if combined therapy could effectively modulate this axis. To the best of our knowledge, this is the first study to evaluate this specific therapeutic combination targeting the purinergic axis in a 3D co-culture model of HCC.

## 2. Materials and Methods

### 2.1. RNA-Seq Data Analysis

Publicly available RNA-sequencing (RNA-seq) data and corresponding clinical information from TCGA-LIHC were retrieved from the Genomic Data Commons (GDC) portal using the *TCGAbiolinks* R package (v2.34.1). We selected samples processed via the “*STAR—Counts*” workflow to obtain raw gene expression counts. The final dataset comprised 421 samples, including 371 primary solid tumors and 50 adjacent solid tissue normal samples. Data were downloaded and prepared into a *SummarizedExperiment* object using the *GDCdownload* and *GDCprepare* functions, respectively. A subset of 24 genes characterizing the human purinergic system was selected for downstream analysis, including P2X receptors (*P2RX1-7*), P2Y receptors (*P2RY1*, *2*, *4*, *6*, *11–14*), adenosine receptors (*ADORA1*, *ADORA2A*, *ADORA2B*), and key ectonucleotidases (*ENTPD1*, *NT5E*, *ADA*, *CD38*, *ALPL*). Gene symbols were mapped to *Ensembl IDs* using the *biomaRt* package (v2.62.1).

Differential expression analysis between primary tumor and normal tissue samples was performed using the *DESeq2* package (v1.46.0). Low-count genes were filtered out prior to analysis to reduce noise. From the initial set of 60,660 genes quantified, low-count genes were filtered out (row sums > 10), resulting in a final set of 48,860 genes for downstream statistical analysis. Raw counts were normalized using the median-ratio method to account for differences in sequencing depth. To ensure the robustness of the findings, we assessed potential batch effects by visually inspecting Principal Component Analysis plots labeled by Tissue Source Site (TSS). No significant batch-driven clustering was observed that would necessitate covariate adjustment in the model design (ANOVA *p* = 0.139). The Wald test was employed to determine statistical significance, and *p*-values were adjusted for multiple comparisons using the Benjamini–Hochberg (FDR) procedure. Genes with an adjusted *p*-value (padj) < 0.05 were considered differentially expressed (DEGs).

To elucidate the biological functions associated with the DEGs, Gene Ontology (GO) and Kyoto Encyclopedia of Genes and Genomes (KEGG) pathway enrichment analyses were conducted using the *clusterProfiler* package (v4.14.6). Over-representation analysis (ORA) was performed, with a primary focus on GO Biological Processes (BP) and KEGG pathways relevant to cancer biology and purinergic signaling. Significance was determined by a hypergeometric test with Benjamini–Hochberg correction (FDR < 0.05). To ensure the biological relevance of the results within the context of HCC, a curation step was applied to prioritize pathways associated with metabolism, signaling transduction, and immune response.

The prognostic value of the 24 purinergic genes was evaluated using the *survival* (v3.6.4) and *survminer* (v0.5.1) packages. Overall survival (OS) data were derived from clinical metadata (*days_to_death* and *days_to_last_follow_up*). For each gene, patients were stratified into “High” and “Low” expression groups based on the median expression value. Survival differences were assessed using the *Kaplan–Meier* method and the log-rank test. Additionally, multivariate Cox proportional hazards regression models were fitted to calculate *Hazard Ratios* (HR) and 95% confidence intervals. *p*-values from Cox models were adjusted for multiple comparisons (FDR), with significance defined as FDR < 0.05.

To investigate functional connectivity changes in the purinergic system during tumorigenesis, gene co-expression networks were constructed separately for normal and tumor tissues. Pairwise Pearson correlation coefficients (r) were calculated for the 24 genes. An undirected graph was constructed using the *igraph* package (v2.1.4), with edges defined by a hard threshold on absolute correlation, |r| > 0.4. Network topology was characterized by global metrics (density and node counts/edges) and node centrality (degree). Networks were visualized using the Fruchterman–Reingold force-directed layout to reveal community structure.

A machine learning approach was implemented to validate the discriminatory power of the purinergic signature and identify robust biomarkers. A Random Forest (RF) classifier was trained to distinguish tumor from normal samples using the *caret* (v7.0.1) and *randomForest* (v4.7.1.2) packages. The dataset was split into training (75%) and testing (25%) sets. The model was trained using 10-fold cross-validation, repeated 5 times, with optimization for the Area Under the ROC Curve (AUC). Model performance was evaluated on the independent test set using metrics including accuracy, sensitivity, specificity, and AUC. To identify the most stable predictors, *Recursive Feature Elimination* (RFE) with Random Forest was performed using 100 bootstrap iterations. Genes were ranked by selection frequency across bootstrap samples.

### 2.2. Cell Culture and Spheroid Formation

The HepG2 hepatocellular carcinoma cell line was obtained from the Cell Bank of Rio de Janeiro (HUCFF, UFRJ, RRID: CVCL_0027). The LX-2 hepatic stellate cell line (RRID: CVCL_5792) was generously provided by Dr. Karen C. Martinez de Moraes, who supplied the cells from UNESP under the authorization of Prof. Scott Friedman. Both cell lines were cultured in low-glucose Dulbecco’s Modified Eagle Medium (Sigma-Aldrich, St. Louis, MO, USA; Cat. No. D5523) supplemented with 10% fetal bovine serum (FBS, Cripion Biotecnologia LTDA, Andradina, SP, Brazil; Cat. No. FB0010SI) and 1% penicillin/streptomycin (Sigma-Aldrich, St. Louis, MO, USA; Cat. No. P0781) at 37 °C in a 5% CO_2_ humidified atmosphere.

To generate HepG2 and HepG2/LX-2 spheroids (1:1 ratio), a total of 3 x 10^4^ viable cells were seeded in 96-well round-bottom plates coated with 1.5% agarose for viability assays. For the remaining assays, Nunclon Sphera 96-well U-bottom ultra-low attachment plates (Thermo Fisher Scientific, Roskilde, Denmark; Cat. No. 174929) were used. After seeding, the plates were centrifuged for 10 min at 1200 rpm to aid spheroid formation.

### 2.3. Treatments

Stock solutions of sorafenib tosylate (Sigma, Cat. No. SML2633; 50 mM) and Doxazosin mesylate (Sigma, Cat. No. D9815; 10 mM) were prepared in dimethyl sulfoxide (DMSO) and stored at −20 °C. For in vitro treatments, drugs were diluted in the culture medium to the desired final concentrations. The final concentration of DMSO in the culture medium was kept below 1% (*v*/*v*) to avoid solvent toxicity.

The treatment groups used in the experiments, after determining the half-maximal inhibitory concentration (IC50), were as follows: vehicle (0.35% DMSO); sorafenib monotherapy at 10 µM and 25 µM; and co-treatment at 10 µM sorafenib + 30 µM doxazosin (S1D) and 25 µM sorafenib + 30 µM doxazosin (S2D).

### 2.4. Cell Viability Assay

Cell viability was evaluated using the CellTiter-Glo^®^ 3D kit (Promega Corporation, Madison, WI, USA; Cat No. G9681). The experiment was performed according to the manufacturer’s instructions. Experiments were performed in three independent biological replicates, with four spheroids per condition in each replicate. The results were then used to calculate the spheroid IC50 values.

### 2.5. Quantitative Real-Time Polymerase Chain Reaction (qPCR)

Total RNA content from spheroids was isolated using TRIzol reagent (Invitrogen, Carlsbad, CA, USA) and quantified by spectrophotometry. Experiments were performed in three/four independent biological replicates, with ten spheroids per condition in each replicate. Complementary DNA (cDNA) was synthesized from 1.4 μg of total RNA using the High-Capacity cDNA Reverse Transcription Kit (Applied Biosystems, Vilnius, Lithuania; Cat. No. 4368814) and stored at −20 °C until use. qPCR was performed in triplicate on a StepOnePlus™ Real-time PCR system (Applied Biosystems, Waltham, MA, USA) using the GoTaq^®^ qPCR Master Mix (Promega Corporation, Fitchburg, WI, USA; Cat. No. A6001), according to the manufacturer’s instructions. The thermal cycling profile for gene expression consisted of an initial denaturation step at 95 °C for 5 min followed by 40 cycles of 15 s at 95 °C, 30 s at 60 °C, and 30 min at 72 °C for data acquisition. All primer sequences used in this study are described in the Supplementary Table S1. Values were normalized to the relative expression levels of the housekeeping gene Receptor for Activated C Kinase 1 (*RACK1*). To select the most accurate and stable reference gene for this analysis, RefFinder, a web-based tool (https://www.ciidirsinaloa.com.mx/RefFinder-master/ (accessed on 30 June 2025)), was used [29]. Relative quantification was calculated using the *2^−ΔΔCt^* method [30].

### 2.6. Flow Cytometry

To determine the expression of CD39, CD73, and ADA proteins after 48 h of treatment, spheroids were collected, washed with phosphate-buffered saline (PBS), and dissociated using TrypLE™ Express reagent (Gibco, Thermo Fisher Scientific, Paisley, UK, Cat No. 12605028). Cells were then stained with BD Pharmingen™ PerCP-Cy™5.5 mouse anti-human CD39 (clone TU66, Cat. No. 564899), BD Pharmingen™ PE mouse anti-human CD73 (clone AD2, Cat. No. 550257), and FITC mouse anti-human CD26 (clone M-A261, Cat. No. 555436), according to the manufacturer’s instructions.

To evaluate active caspase-3 expression, dissociated spheroid cells were permeabilized and stained with BD Pharmingen™ PE rabbit anti-active caspase-3 antibody (clone C92-605, Cat. No. 550821). Experiments were performed in three/four independent biological replicates, with six spheroids per condition in each replicate. Protein expression levels were quantified as the fold change of median fluorescence intensity (MFI). For sample acquisition and result analysis, the BD Accuri™ cytometer with C6 Plus software (version 1.0.23.1; BD Biosciences, San Jose, CA, USA) was used. The results were analyzed using FlowJo^®^ v10 software (FlowJo LLC, Ashland, OR, USA).

### 2.7. Statistical Analysis

All statistical analyses and graphical representations were performed using the Rstudio (v4.4.3) with the *rstatix* (v0.7.3), *ggpubr* (v0.6.1), and *ggplot2* (v4.0.1) packages. Data distribution and homogeneity of variances were assessed using Shapiro–Wilk and Levene’s tests, respectively. For datasets satisfying parametric assumptions, multiple comparisons were performed using One-Way ANOVA followed by Tukey’s HSD post hoc test. To evaluate the influence of the tumor microenvironment on pharmacological response, a Two-Way ANOVA was employed to assess the interaction between ‘Treatment’ and ‘Culture System’ (HepG2 vs. HepG2/LX-2).

## 3. Results

### 3.1. RNA-Seq Analysis

#### 3.1.1. Adenosine-Related Enzymes and Receptors Are Upregulated in HCC

**Figure 1:**
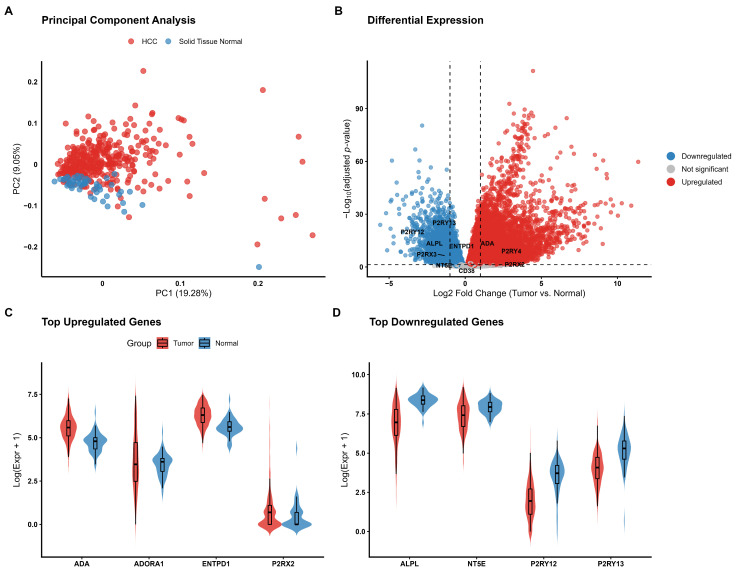
Transcriptional landscape of the purinergic signaling system in Hepatocellular Carcinoma (HCC). (**A**) Principal Component Analysis (PCA) based on the expression profiles of 24 purinergic genes distinguish primary tumor samples (red, n = 371) from adjacent normal tissue samples (blue, n = 50). PC1 accounts for 19.28% of the total variance. (**B**) Volcano plot illustrating differential expression analysis (Tumor vs. Normal). Red points represent significantly upregulated genes (log2FC > 0, FDR < 0.05), blue points represent downregulated genes (log2FC < 0, FDR < 0.05), and gray points indicate non-significant genes. Key purinergic genes are labeled. (**C**,**D**) Violin plots comparing the expression distribution (log-transformed) between adjacent normal tissue samples (Blue) and primary tumor (Red) samples. Panel (**C**) displays the top upregulated genes, while Panel (**D**) shows the top downregulated genes. Internal boxplots represent the median and interquartile range. The violin’s width represents the density of samples at different expression levels.

To investigate the transcriptional landscape of the purinergic system in HCC, we analyzed RNA-seq data from 421 samples. Principal component analysis (PCA) was performed as an exploratory step to visualize the variation patterns within the purinergic gene set. The analysis showed a visual separation between tumor and normal samples (Figure 1A). However, this clustering should be interpreted with caution, as it minimizes group separation based on a pre-defined subset of functionally related genes rather than providing independent evidence of global transcriptomic differences. Nevertheless, the pattern suggests that the coordinated expression of these 24 genes is altered in the tumor context. Differential expression analysis identified significant alterations in the purinergic signaling network (Figure 1B). The expression pattern was heterogeneous: genes encoding key enzymes for ATP and adenosine metabolism, such as *ENTPD1* (CD39, log2FC = 0.64) and *ADA* (log2FC = 0.87), as well as adenosine receptors *ADORA1* and *ADORA2A*, were significantly upregulated in HCC (Figure 1B). Furthermore, receptors such as *P2RX2* and *P2RY4* exhibited the highest magnitude of upregulation. Conversely, genes associated with ADP signaling, particularly *P2RY12* (log2FC = −2.42) and *P2RY13* (log2FC = −1.92), were among the most downregulated genes in the tumor tissue (Figure 1B). *NT5E* (CD73), responsible for adenosine generation, was downregulated (log2FC = −0.79), suggesting a complex imbalance in the extracellular nucleotide hydrolysis chain. Analysis of individual gene distributions confirmed robust overexpression of *ADA*, *ENTPD1*, *P2RX2*, and *ADORA1* (Figure 1C) and significant downregulation of *P2RY12*, *P2RY13*, *NT5E*, and *ALPL* in tumor samples (Figure 1D). Comprehensive statistical results for all 24 analyzed genes are provided in Table 1.

#### 3.1.2. Functional Enrichment Links the Purinergic Signature to Cell Survival and Immune Response Pathways

**Figure 2:**
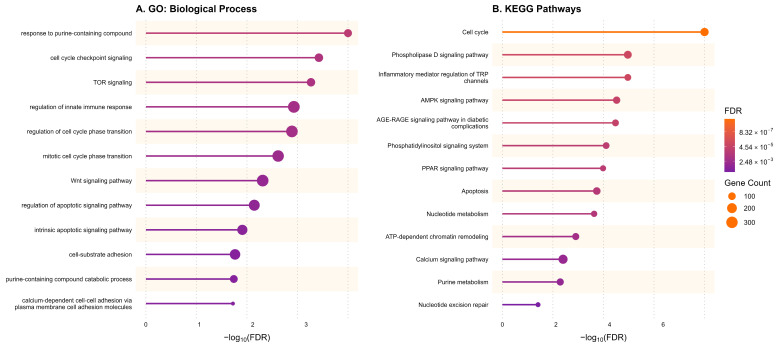
Functional enrichment analysis of purinergic genes in HCC. (**A**) Lollipop plot showing the top enriched Gene Ontology (GO) Biological Processes. (**B**) Lollipop plot showing the top enriched KEGG pathways. The length of the line and the color of the dot represent statistical significance (−log10 FDR). The size of the dot represents the number of differentially expressed genes (Gene Count) associated with each term.

Analysis of Gene Ontology (GO) Biological Processes revealed that the differentially expressed genes are significantly enriched in functions related to nucleotide signaling and metabolism, such as “response to purine-containing compound” and “purine-containing compound catabolic process” (Figure 2A). Furthermore, there was a strong enrichment of pathways governing cell fate and survival, including “regulation of apoptotic signaling”, “Wnt signaling pathway”, and “TOR signaling”. Notably, terms related to the “regulation of innate immune response” were also significant, highlighting the immunomodulatory role of this system.

Complementary KEGG pathway analysis corroborated these findings (Figure 2B). The “Purine metabolism” and “Nucleotide metabolism” pathways were directly enriched, confirming the functional impact of the gene signature. The analysis linked purinergic alterations to major oncogenic signaling cascades, including the “AMPK signaling pathway”, “Calcium signaling pathway”, and “Phospholipase D signaling pathway”. The “Cell cycle” and “Apoptosis” pathways were among the most significant hits (lowest FDR), reinforcing the connection between purinergic signaling and proliferative and viability profiles of HCC.

#### 3.1.3. Prognostic Value of Purinergic Gene Expression in HCC

To evaluate the clinical relevance of the purinergic system, we assessed the association between expression levels of the 24 purinergic genes and patient overall survival (OS). Univariate Cox proportional hazards regression models were first used to identify prognostic genes (FDR < 0.05), followed by multivariate Cox regression analysis adjusted for patient age and pathological tumor stage to determine whether these genes were independent prognostic factors. Notably, high expression of *P2RY6*, *ADA*, *NT5E*, *P2RX4*, *P2RY4*, and *ADORA1* was consistently associated with a higher risk of death (Hazard Ratio > 1; Supplementary Table S2), indicating a poor prognosis for patients with elevated levels of these transcripts.

**Figure 3:**
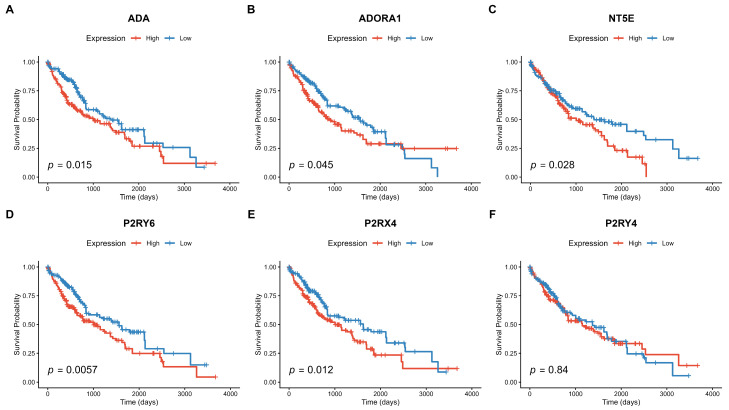
Kaplan–Meier survival curves for the six genes significantly associated with overall survival (FDR < 0.05). Patients were stratified into “High” (red) and “Low” (blue) expression groups based on the median gene expression value. The *p*-values shown are from the log-rank test. High expression of (**A**) *ADA*, (**B**) *ADORA1*, (**C**) *NT5E*, (**D**) *P2RY6*, and (**E**) *P2RX4* is significantly associated with poor prognosis (shorter survival time). (**F**) Although *P2RY4* was significant in multivariate Cox regression, it did not show significant stratification in Kaplan–Meier analysis using a median split (*p* = 0.84).

Kaplan–Meier survival analysis further confirmed these findings. Patients stratified into the “High Expression” group (above the median) for these six genes had significantly shorter overall survival than those in the “Low Expression” group (Figure 3). *P2RY6* showed the strongest prognostic association (FDR = 8.11 × 10^−8^), followed by *ADA* (FDR = 6.52 × 10^−5^) and *NT5E* (FDR = 0.004). Although *P2RY4* showed a significant association with survival in the continuous Cox regression model (FDR = 0.0095), the Kaplan–Meier analysis using a median split did not reveal a significant difference between high and low expression groups (*p* = 0.84), suggesting that the prognostic impact might be non-linear or driven by a specific subset of patients.

#### 3.1.4. Purinergic Co-Expression Network in HCC

**Figure 4:**
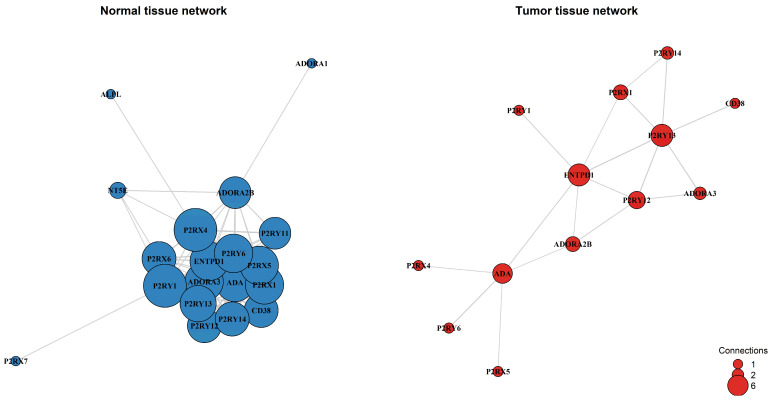
Gene co-expression networks were constructed for normal adjacent tissue and primary tumor tissue. Nodes represent purinergic genes, with node size proportional to the number of connections (degree). Edges represent strong co-expression links (|r| > 0.4). The analysis reveals a transition from a dense, highly integrated network in normal tissue (Density: 0.602; 103 edges) to a fragmented architecture in the tumor (Density: 0.231; 18 edges).

To investigate whether the functional coordination of the purinergic system is remodeled during tumorigenesis, we constructed and compared gene co-expression networks for normal and tumor tissues. This analysis revealed a loss of connectivity and a structural collapse of the purinergic signaling network (Figure 4). In normal liver tissue, the system operates as a highly cohesive module with a network density of 0.602, maintained by 103 strong co-expression links among 19 genes. In contrast, the tumor network exhibited fragmentation, with network density plummeting to 0.231 (a ~61% reduction) and the number of strong connections dropping to just 18. Six genes (*NT5E*, *P2RX6*, *P2RY11*, *ADORA1*, *ALPL*, *P2RX7*) were completely decoupled from the main network in tumor tissue.

A distinct shift in network centrality accompanied the loss of connectivity. In normal tissue, the receptors *P2RX4* and *P2RY1* acted as hubs, each coordinating with 16 other genes. However, these receptors lost their central role, retaining only a single connection in tumor tissue. Conversely, a smaller and weaker core emerged in the tumor, centered around *ENTPD1*, *P2RY13*, and *ADA*, which became the most connected nodes (degree = 5–6). *NT5E* (CD73) became isolated in the tumor network, suggesting a functional uncoupling of the adenosine production machinery.

#### 3.1.5. Predictive Analysis and Biomarker Selection

**Figure 5:**
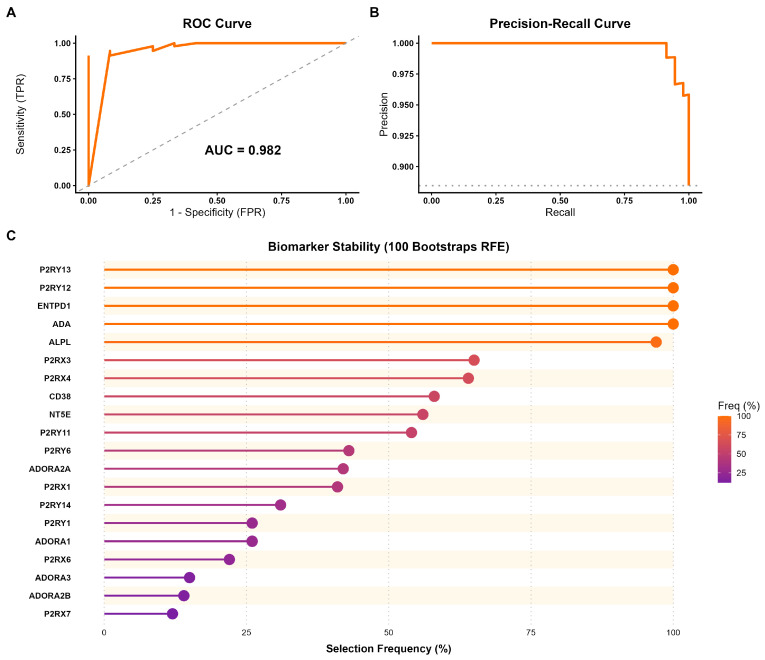
Predictive performance and biomarker stability of the purinergic gene signature in HCC. (**A**) Receiver Operating Characteristic (ROC) curve evaluated on the independent test set. The Random Forest model achieved an Area Under the Curve (AUC) of 0.982, indicating strong discrimination between tumor and normal tissues. (**B**) Precision–Recall (PR) curve showing high precision across recall levels, confirming the model’s robustness despite the dataset’s class imbalance. (**C**) Biomarker stability analysis using Recursive Feature Elimination (RFE) with 100 bootstrap iterations. The lollipop plot illustrates the frequency with which each gene was selected as a key predictor across resampling. Genes are ordered by stability and color-coded by their selection frequency.

To determine whether the transcriptional profile of the 24 purinergic genes constitutes a robust molecular signature capable of distinguishing HCC from normal tissue, we implemented a machine learning approach using a Random Forest classifier. The purinergic signature demonstrated discriminatory power on the test set (25% of the cohort). The model achieved an AUC of 0.982, indicating near-perfect ability to distinguish tumor from normal samples across all classification thresholds (Figure 5A). The precision–recall (PR) curve showed high precision across all recall levels, confirming the model’s robustness even under class imbalance (Figure 5B; Supplementary Table S3).

The model achieved 100% sensitivity, correctly identifying all tumor samples in the test set. The overall accuracy was 93.3% (95% CI: 0.86–0.97). The Balanced Accuracy of 70.8% indicates that the signature’s predictive value exceeds chance (Kappa = 0.558). To refine the signature and identify the most critical biomarkers, we performed RFE with 100 bootstrap iterations. This analysis revealed a hierarchy of predictor stability. A core set of five genes (i.e., *P2RY13*, *P2RY12*, *ENTPD1*, *ADA*, and *ALPL*) was selected in nearly 100% of the bootstrap iterations (Figure 5C).

### 3.2. Cytotoxicity and Apoptotic Effect of Combined Therapy in Spheroids

**Figure 6:**
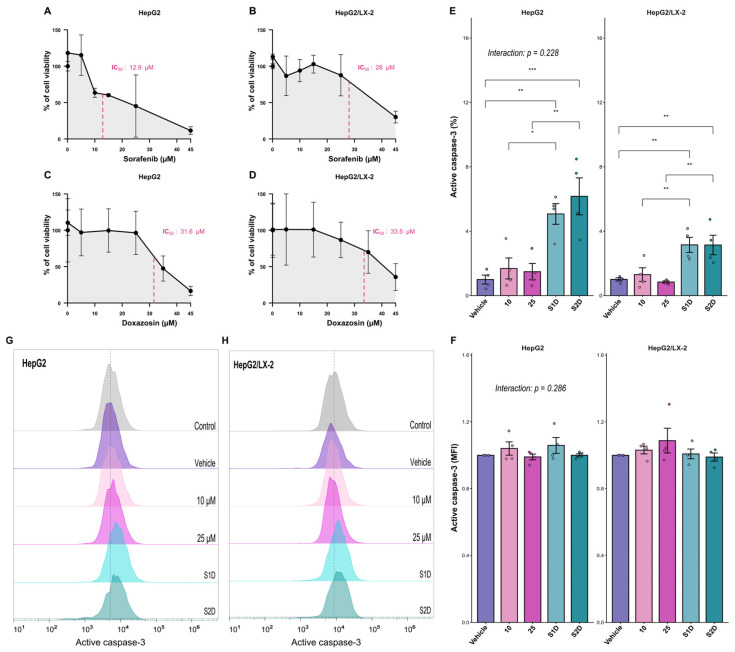
Cytotoxicity and apoptotic efficacy of sorafenib and doxazosin combination in 3D liver tumor models. (**A**–**D**) Concentration-response viability curves determining the half-maximal inhibitory concentration (IC50) of sorafenib (**A**,**B**) and doxazosin (**C**,**D**) in HepG2 mono-culture and HepG2/LX-2 co-culture spheroids. (**E**–**H**) Evaluation of apoptosis via active caspase-3 detection by flow cytometry. (**E**) Quantification of caspase-3 expression intensity (MFI fold change) and (**F**) frequency of positive cells (%) in spheroids treated with Sorafenib monotherapy (10 µM and 25 µM) or combined with doxazosin 30 µM (S1D: Sorafenib 10 µM + Doxazosin; S2D: Sorafenib 25 µM + Doxazosin). (**G**,**H**) Representative flow cytometry histograms showing the fluorescence shift of active caspase-3 in HepG2 (**G**) and HepG2/LX-2 (**H**) spheroids. Data represent mean ± SD of four independent experiments. Statistical significance was determined by ANOVA, * *p* < 0.05, ** *p* < 0.01, *** *p* < 0.001. Interaction *p*-values indicate the interplay between treatment and culture system.

To determine the optimal therapeutic concentrations for the 3D models, we first established the IC50 of sorafenib and doxazosin. The IC50 of sorafenib was 12.9 µM in HepG2 spheroids and increased to 28.0 µM in HepG2/LX-2 spheroids, highlighting the protective role of the stromal component (Figure 6A,B). The IC50 for doxazosin was approximately 30 µM in both types of spheroids (Figure 6C,D). Based on these results, we chose sublethal concentrations of sorafenib (10 and 25 µM) and combined them with a fixed concentration of doxazosin (30 µM) for subsequent assays.

We evaluated whether doxazosin could enhance sorafenib-induced cell death by assessing active caspase-3 staining. In HepG2 spheroids, the combined treatment (S1D and S2D) significantly increased apoptosis compared with vehicle or single treatments (*p* = 0.0055 and *p* = 0.0005, respectively) (Figure 6E–H). In HepG2/LX-2 spheroids, the potentiating effect was preserved but lower than in HepG2 spheroids (S1D *p* = 0.0519 and S2D *p* = 0.0082) and still significant compared to the vehicle (*p* = 0.0040 for both co-treatments) (Figure 6E–H). These results validate the sorafenib plus doxazosin combination as an effective strategy to overcome the intrinsic resistance of multilayer 3D spheroids.

### 3.3. Modulation of Purinergic Signaling Pathway Gene Expression in Spheroids

**Figure 7:**
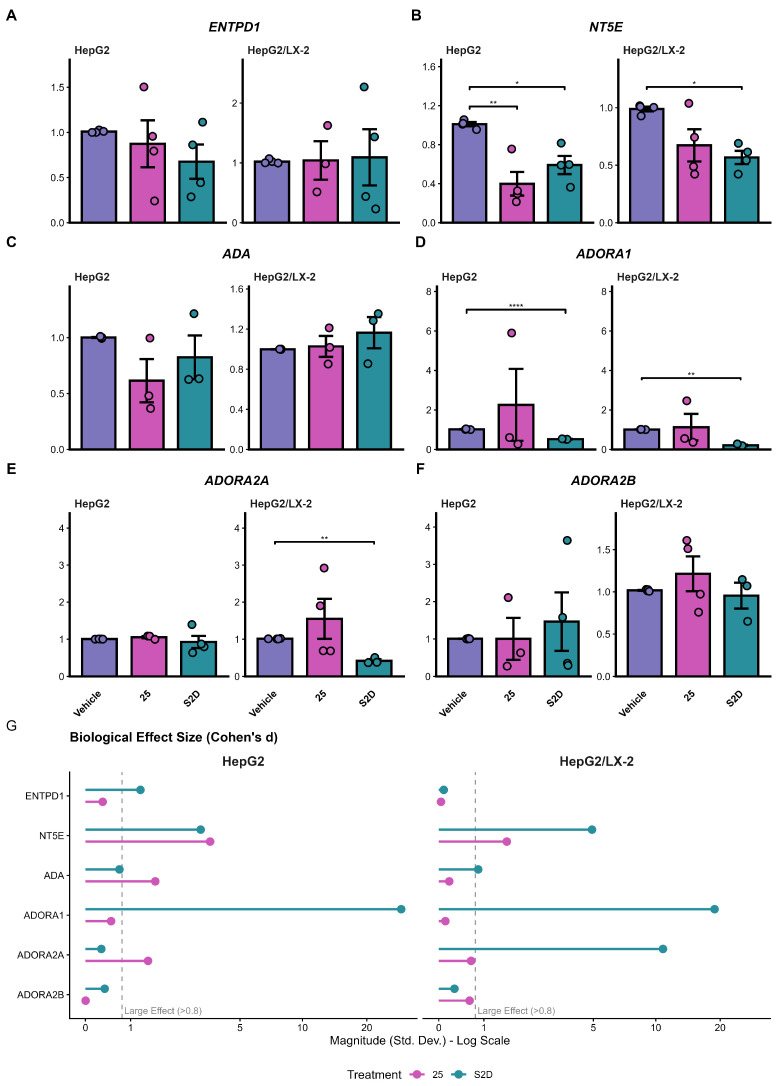
Purinergic signaling pathway gene expression by qPCR. (**A**–**F**) Relative mRNA expression of ecto-enzymes (*ENTPD1*, *NT5E*, *ADA*) and adenosine receptors (*ADORA1*, *ADORA2A*, *ADORA2B*) in HepG2 and HepG2/LX-2 spheroids treated with 25 µM of sorafenib or S2D. Data were normalized to the housekeeping gene RACK1 and are expressed as mean ± SD of three/four independent experiments, * *p* < 0.05, ** *p* < 0.01, **** *p* < 0.0001. (**G**) Analysis of biological effect size (Cohen’s d); the dashed line indicates the threshold for a large effect (>0.8), highlighting biological relevance independent of *p*-value.

To expand the study of purinergic signaling, we analyzed the expression of key enzymes (*ENTPD1*, *NT5E*, *ADA*) and adenosine receptors (*ADORA1*, *ADORA2A*, *ADORA2B*) by qPCR in HepG2 and HepG2/LX-2 spheroids treated with 25 µM of sorafenib (“25”) or the cotreatment S2D (Figure 7). The expression of *ENTPD1* remained unaltered across all conditions and culture models (Figure 7A). In contrast, *NT5E* (CD73) was significantly downregulated. In HepG2 spheroids, both treatments reduced *NT5E* expression (*p* < 0.01 for 25 µM; *p* < 0.05 for S2D). In the co-culture model, only S2D maintained efficacy, significantly reducing *NT5E* levels (*p* < 0.05) with a large effect size (Cohen’s d > 3.0, Figure 7G), suggesting robust inhibition of adenosine production capacity (Figure 7B).

*ADA* expression presented a downward trend in HepG2 spheroids treated with 25 µM of sorafenib (Figure 7C). Although classical statistical significance was not reached due to sample variability (*p* > 0.05), the analysis of biological magnitude revealed a large effect size (Cohen’s d = 1.63; Figure 7G), suggesting a biologically relevant modulation. No changes were observed in the co-culture model for *ADA*.

Finally, we assessed the downstream receptors. *ADORA1* expression was suppressed by S2D cotreatment in both mono- and co-cultures (*p* < 0.0001 and *p* < 0.01; Figure 7D), with massive effect sizes observed in the co-culture model (Figure 7G). *ADORA2A* exhibited a specific response in the co-culture setting: while the 25 µM treatment showed high heterogeneity, S2D treatment significantly downregulated *ADORA2A* expression (*p* < 0.01) in the presence of LX-2 cells (Figure 7E). The expression levels of *ADORA2B* receptors remained stable across all experimental conditions (Figure 7F).

### 3.4. Purinergic Signaling Flow Cytometry

**Figure 8:**
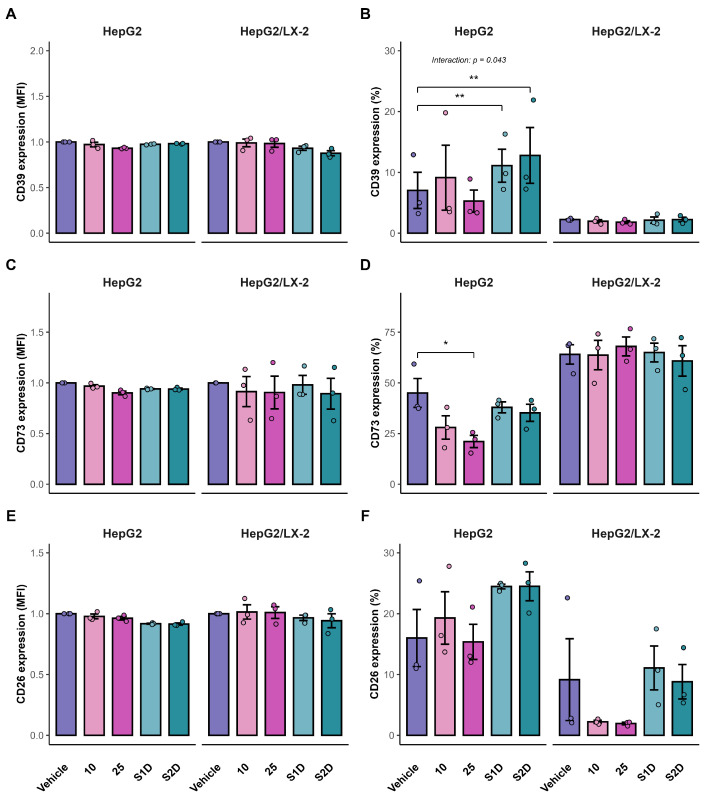
Surface protein expression of purinergic ecto-enzymes in HepG2 spheroids. Flow cytometry analysis of CD39 (**A**,**B**), CD73 (**C**,**D**), and CD26 (**E**,**F**) in HepG2 monocultures and HepG2/LX-2 co-cultures. Panels A, C, and E show the Median Fluorescence Intensity (MFI) fold change relative to vehicle control. Panels (**B**,**D**,**F**) show the frequency (%) of positive cells. Data represent mean ± SD of three independent experiments, * *p* < 0.05, ** *p* < 0.01.

To evaluate whether the transcriptional changes in purinergic signaling translated to cell-surface protein expression, as well as to assess the influence of the tumor microenvironment on drug response, the expression of CD39, CD73, and CD26 was analyzed in mono- and co-culture spheroids by flow cytometry. In HepG2 spheroids, combined sorafenib and doxazosin treatment significantly increased the percentage of CD39^+^ cells compared with the vehicle group (Sf: *p* = 0.007; S2D: *p* = 0.008; Figure 8B). This increase was observed in the co-culture system, as indicated by a significant Treatment × System interaction (Two-way ANOVA, *p* = 0.043). The CD39 MFI remained unchanged after doublet exclusion (Figure 8A).

For CD73, high concentration of sorafenib (25 µM) significantly reduced the proportion of CD73^+^ cells in HepG2 spheroids (*p* = 0.036; Figure 8D), whereas no significant differences were detected in the co-culture model (*p* > 0.05). CD73 MFI did not vary across conditions in either culture system (Figure 8C). CD26 expression remained stable in both frequency and intensity under all treatments and in both mono- and co-culture models (Figure 8E,F), indicating limited responsiveness of this marker to acute pharmacological modulation at the protein level.

## 4. Discussion

In this study, we propose integrating transcriptomic analysis with multicellular spheroids composed of HCC cells and HSCs to mimic the tumor microenvironment and its complexity. This finding underscores the potential of using a mixed spheroid model comprising tumor and stromal cells (HepG2/LX-2) to study, in vitro, the efficacy of therapeutic interventions. This model offers a valuable representation of the in vivo tumor environment, facilitating the exploration of therapeutic strategies. Furthermore, evidence has emerged that the combination of doxazosin and sorafenib can modulate purinergic signaling. Additionally, it has been demonstrated that stromal cells present in spheroids influence the response to treatment, leading to increased resistance.

Given the existing evidence that the purinergic system plays a central role in the development of therapeutic resistance in other types of cancer [21,24], we investigated the gene expression profile of purinergic genes in HCC. Genes related to ATP metabolism *(ENTPD1*, *P2RX2*, and *P2RY4*) and adenosine receptors (*ADORA1* and *ADORA2A*) appeared to be upregulated. For example, it has been demonstrated that ADORA2A receptors can activate PI3K-Akt signaling, promoting the proliferation and invasion of HCC cells; these pathways are typically activated in sorafenib-resistant cells [13,30]. This scenario is supported by analyses using GO and KEGG, which identified enriched pathways in our dataset, indicating that the purinergic system modulates tumor growth and survival pathways. Surprisingly, the *NT5E* gene was among the top downregulated genes. High *NT5E* gene expression (CD73 protein) is typically associated with therapy resistance and a worse prognosis in HCC [23,30]. *NT5E* is highly expressed in hepatic stellate cells and may appear downregulated in this dataset because the samples included the entire tumor tissue of patients, which is primarily composed of tumor hepatocytes [31,32].

Beyond differential expression, co-expression network analysis revealed a substantial rewiring of the purinergic system during hepatocarcinogenesis. In normal liver tissue, purinergic genes formed a highly interconnected network, consistent with coordinated, homeostatic regulation of extracellular ATP and adenosine signaling. In contrast, tumor tissue exhibited a pronounced loss of connectivity and network density, reflecting a breakdown of coordinated physiological programs and a shift toward simplified survival-oriented signaling, a phenomenon commonly observed during malignant transformation [33,34]. This reorganization was accompanied by changes in network centrality. While *P2RX4* and *P2RY1* acted as major hubs in normal tissue, reflecting their constitutive role in maintaining biliary homeostasis and hepatic regeneration [35], these receptors lost connectivity in tumors. In contrast, metabolic enzymes such as *ENTPD1* (CD39) and *ADA* emerged as central nodes. The increased centrality of *ADA* is consistent with our prognostic findings and supports a model in which HCC cells prioritize controlling extracellular adenosine levels to promote immune evasion and metabolic adaptation to hypoxia [36]. Notably, *NT5E* (CD73) was isolated within the tumor network despite its known pro-tumoral role, a finding that can be explained by its predominant expression in stromal compartments, particularly in activated hepatic stellate cells and cancer-associated fibroblasts, rather than in tumor hepatocytes [24,37].

The network reprogramming identified in this study was further supported by survival analysis, which demonstrated that high expression of key purinergic components is associated with poor overall survival. Upregulation of genes in the adenosinergic axis (i.e., *NT5E*, *ADA*, and *ADORA1*) correlated with an unfavorable prognosis, reinforcing the notion that adenosine signaling constitutes an active pro-tumoral mechanism rather than a passive consequence of tumor hypoxia. This clinical pattern is consistent with extensive evidence from other solid tumors, including breast and colorectal cancers, where enhanced CD73-mediated adenosine production and adenosine receptor activation promote immune evasion, metastatic dissemination, and resistance to therapy [37,38]. In our study, high *ADORA1* expression correlated with poor overall survival, consistent with its reported tumor-promoting functions across several solid malignancies, where *ADORA1* signaling supports proliferation and survival through activation of oncogenic pathways such as AKT and MAPK [39,40]. The most robust prognostic predictor identified was the receptor *P2RY6*. Although underexplored in the hepatic context, its clinical relevance in HCC was recently supported by Chen et al. (2025) [40], who identified *P2RY6* as a core component of an adenosine metabolism-related gene signature, in which elevated *P2RY6* expression was associated with poor prognosis, enhanced immune infiltration, and increased expression of immune checkpoint molecules in HCC. This association is consistent with reports in gastric cancer, where *P2RY6* activation suppresses tumor cell proliferation, underscoring the context-dependent nature of purinergic signaling across tumor types [41].

Using a machine learning framework, we assessed whether the expression profile of purinergic genes alone could distinguish hepatocellular carcinoma from normal liver tissue. The Random Forest model achieved high discriminatory performance (AUC = 0.982), indicating that purinergic remodeling represents a consistent transcriptomic feature of HCC. However, this result should be interpreted with caution due to the class imbalance in the TCGA dataset. Rather than proposing a diagnostic classifier, these findings function as an in silico validation of the biological relevance of purinergic reprogramming identified in our differential expression and network analyses. Feature selection using bootstrap recursive feature elimination revealed a stable core of the genes *P2RY13*, *P2RY12*, *ENTPD1* (CD39), ADA, and *ALPL* that consistently contributed to tumor classification. The prominence of *P2RY12* and *P2RY13* is biologically meaningful, as these ADP-responsive receptors are typically associated with homeostatic purinergic signaling, including platelet regulation and immune cell motility [42,43]. Their reduced expression and strong predictive value align with our network results, which show loss of coordinated receptor signaling and suggest impairment of physiological P2Y12-dependent immune surveillance mechanisms, as previously described in myeloid cells and microglia [44]. In contrast, the recurrent selection of *ENTPD1* and *ADA* reinforces the shift toward an adenosine-enriched extracellular milieu. This metabolic reprogramming is a potential driver of malignancy in HCC, where the overexpression of CD39 (*ENTPD1*) has been independently validated as a mechanism that facilitates tumor invasion and predicts poor overall survival [45,46].

The computational identification of *NT5E*, *ADA*, and *ADORA1* as prognostic biomarkers provided a basis for therapeutic evaluation. Specifically, the network topology analysis suggested that the generation of an adenosine-mediated immunosuppressive state is likely not an intrinsic property of the hepatocyte alone, but relies on a disrupted interplay with the stromal compartment. To bridge the gap between this global transcriptomic landscape and clinical applicability, we transitioned from in silico profiling to a biologically relevant in vitro model. We hypothesized that combining sorafenib with doxazosin, a drug known to interfere with survival pathways downstream of purinergic receptors, could effectively remodel this dysregulated axis. Therefore, we used 3D multicellular spheroids incorporating HSCs (LX-2) to recapitulate the physical and metabolic barriers of the tumor microenvironment, allowing us to investigate whether this pharmacological strategy could overcome stromal-mediated resistance and reverse the expression of the poor-prognosis purinergic gene signature. To this end, we first determined the IC50 values for sorafenib and doxazosin. Notably, the IC50 of sorafenib was higher for HepG2/LX-2 spheroids than for HepG2 spheroids. The same pattern of resistance has been reported in previous studies, such as Khawar et al. (2018) and Song et al. (2016) [6,10]. We evaluated the expression of active caspase-3 and found that both co-treatments (S1D and S2D) promoted apoptosis in the spheroids; however, the level of apoptosis was higher in the HepG2 spheroids. This can be explained by the fact that HSCs act as CAFs in the tumor microenvironment, promoting chemoresistance by secreting factors such as TGF-β and HGF, which induce epithelial–mesenchymal transition (EMT) and tumor growth pathways [24,47]. Additionally, HSCs secrete extracellular matrix proteins, such as type I collagen, which may help form a physical barrier that impairs drug penetration into the spheroids [11].

We evaluated how our treatments could modulate purinergic signaling in spheroids and found that S2D co-treatment decreased the expression of genes associated with poor prognosis, such as *NT5E* and *ADORA1*, in both HepG2 and HepG2/LX-2 spheroids. Additionally, co-treatment decreased *ADORA2A* expression in HepG2/LX-2 spheroids. *ADORA2A* receptors regulate the proliferation and fibrogenesis pathways of HSCs, stimulating the synthesis of types I and III collagen [47]. This explains why co-culture spheroids are more resistant to sorafenib treatment. Furthermore, activation of these receptors mediates the phosphorylation of the PI3K/Akt pathway, inducing HCC cell proliferation and invasion [31]. In this scenario, doxazosin was able to attenuate this resistance by promoting caspase-3-mediated apoptosis in HepG2/LX-2 spheroids. These data suggest that doxazosin acts not only as a cytotoxic agent but also as a molecular modulator that disrupts purinergic survival signaling, potentially resensitizing tumor cells to sorafenib. However, the translation from transcriptional modulation to protein-level changes revealed significant mechanisms of resistance. While S2D treatment effectively reduced *NT5E* mRNA levels, it failed to significantly decrease the frequency of CD73^+^ cells in the co-culture model. This suggests that the stromal turnover of CD73 protein is highly stable or that post-transcriptional compensatory mechanisms maintain its surface expression despite transcriptional inhibition. Furthermore, we observed a paradoxical increase in the frequency of CD39^+^ cells following treatment. This phenomenon likely represents a compensatory feedback loop, wherein tumor cells upregulate the upstream enzyme (CD39) in response to the blockade of the downstream pathway, constituting a common adaptive response observed in metabolic signaling networks [23]. These discrepancies between mRNA and protein dynamics underscore the adaptability of the purinergic system and indicate that, although the sorafenib–doxazosin combination is effective, complete inhibition of the adenosinergic axis may require direct enzymatic inhibition of CD73, combined with transcriptional modulation.

Finally, it is important to acknowledge the limitations regarding the ‘normal’ control samples from the TCGA-LIHC cohort. These samples are derived from tumor-adjacent tissue and may not represent transcriptionally healthy liver; rather, they often reflect a microenvironment of chronic inflammation or cirrhosis, which are common precursors to HCC. Therefore, the differential expression observed here likely captures differences between malignant tissue and a chronically diseased liver background, rather than purely healthy tissue. However, given that most HCC cases develop in the context of fibrosis or cirrhosis, identifying markers that distinguish the tumor from this altered background remains clinically relevant. Our study successfully identifies key purinergic targets involved in therapy resistance; however, it is limited by the in vitro nature of the models and the use of established cell lines. While the 3D co-culture effectively recapitulates stromal protection, it lacks the systemic complexity of a living organism. Consequently, future translational steps should focus on validating the re-sensitization potential of doxazosin in in vivo HCC models and confirming the prognostic stability of the purinergic signature in independent clinical cohorts. These findings establish a solid framework for further exploring this combination therapy in more complex preclinical settings.

## 5. Conclusions

In summary, we demonstrated that the signature of dysregulated purinergic genes may be associated with poor prognosis in patients with HCC in silico and that sorafenib treatment with doxazosin can attenuate the expression of dysregulated genes via the adenosine axis in vitro. Moreover, our findings indicate that the co-culture spheroid model offers a more representative system for investigating potential cancer therapies targeting the purinergic system, accounting for the role of stromal cells in purinergic signaling and therapy resistance.

## Figures and Tables

**Table 1:** Differential expression statistics for the 24 purinergic system genes in HCC versus normal tissue.

Gene Symbol	Log2 Fold Change	Adj. *p*-Value (FDR)	Regulation Status
*P2RX2*	2.71	<0.001	Upregulated
*P2RY4*	2.49	<0.001	Upregulated
*ADORA1*	1.56	<0.001	Upregulated
*P2RX5*	0.96	0.002	Upregulated
*ADA*	0.87	<0.001	Upregulated
*P2RY6*	0.82	<0.001	Upregulated
*ADORA2A*	0.8	<0.001	Upregulated
*ENTPD1*	0.64	<0.001	Upregulated
*P2RX4*	0.62	<0.001	Upregulated
*ADORA2B*	0.58	0.026	Upregulated
*P2RY11*	0.36	0.004	Upregulated
*CD38*	−0.56	0.037	Downregulated
*P2RY1*	−0.58	<0.001	Downregulated
*NT5E*	−0.79	<0.001	Downregulated
*P2RX3*	−1.31	<0.001	Downregulated
*ALPL*	−1.58	<0.001	Downregulated
*P2RY13*	−1.92	<0.001	Downregulated
*P2RY12*	−2.42	<0.001	Downregulated
*P2RY14*	0.36	0.087	Not significant
*P2RY2*	0.32	0.077	Not significant
*P2RX7*	−0.26	0.266	Not significant
*ADORA3*	−0.26	0.278	Not significant
*P2RX1*	−0.08	0.758	Not significant
*P2RX6*	−0.08	0.826	Not significant

## Data Availability

The datasets used and analyzed during the current study are available from the corresponding author on reasonable request.

## Supplementary Materials

The following supporting information can be downloaded at: https://www.mdpi.com/article/10.3390/metabo16030152/s1, Table S1: Primers sequences; Table S2: Survival analysis stats, Table S3: Random Forest classifier stats.

## Abbreviations

The following abbreviations are used in this manuscript:

2D	Two-dimensional
3D	Three-dimensional
ADA	Adenosine deaminase
ALPL	Alkaline phosphatase, liver/bone/kidney
AUC	Area under the ROC curve
BP	Biological processes
CAFs	Cancer-associated fibroblasts
cDNA	Complementary DNA
DEGs	Differentially expressed genes
DMSO	Dimethyl sulfoxide
ECM	Extracellular matrix
EMT	Epithelial–mesenchymal transition
*ENTPD1*	Ectonucleoside triphosphate diphosphohydrolase 1 (ntpdase-1)
FBS	Fetal bovine serum
GDC	Genomic data commons
GO	Gene ontology
HCC	Hepatocellular carcinoma
HR	Hazard ratio
HSCs	Hepatic stellate cells
IC50	Half-maximal inhibitory concentration
KEGG	Kyoto Encyclopedia of Genes and Genomes
MASLD	Metabolic-associated steatotic liver disease
MCTS	Multicellular tumor spheroids
MFI	Median fluorescence intensity
*NT5E*	Ecto-5′-nucleotidase
ORA	Over-representation analysis
OS	Overall survival
PBS	Phosphate-buffered saline
PCA	Principal component analysis
PI3K	Phosphoinositide 3-kinase
PR	Precision–recall
qPCR	Quantitative real-time polymerase chain reaction
*RACK1*	Receptor for activated C kinase 1
RF	Random forest
RFE	Recursive feature elimination
RNA-seq	RNA sequencing
TCGA	The cancer genome atlas
TCGA-LIHC	The cancer genome atlas–liver hepatocellular carcinoma
TME	Tumor microenvironment
TSS	Tissue Source Site
